# Tim1 and Tim3 are not essential for experimental allergic asthma

**DOI:** 10.1111/j.1365-2222.2011.03728.x

**Published:** 2011-07

**Authors:** J L Barlow, S H Wong, S J Ballantyne, H E Jolin, A N J McKenzie

**Affiliations:** 1 MRC Laboratory of Molecular BiologyCambridge, UK; 2 MedImmune, Granta ParkCambridge, UK; 3 Department of Biology and Hull York Medical School, Centre for Immunology and Infection, University of YorkYork, UK

**Keywords:** airway hyperreactivity, asthma, eosinophils, Tim1, Tim3, type-2

## Abstract

**Background:**

Initial studies suggested that polymorphisms in *Tim1* and *Tim3* contribute to the development of airway hyperreactivity (AHR) in an acute mouse model of asthma. This was also mirrored in human genetic studies where polymorphisms in *Tim1* and *Tim3* have been associated with atopic populations.

**Objective:**

Further studies using anti-Tim1 or -Tim3 antibodies, or Tim fusion proteins, have also suggested that these molecules may function as regulators of type-1 and type-2 immunity. However, their role in the development of AHR and airway inflammation remains unclear. Given the proposed roles for Tim1 and Tim3 in type-1 and type-2 responses, we sought to determine whether these molecules were important in regulating antigen-driven lung allergy and inflammation.

**Method:**

We used Tim1- and Tim3-deficient mice and determined how the development of allergic lung inflammation was affected.

**Results:**

AHR was induced normally in the absence of both Tim1 and Tim3, although Tim1-deficient mice did show a small but significant decrease in cell infiltration in the lung and blood eosinophilia. Although Tim3 was expressed on CD4^+^ T cells in the allergic lung, Tim1 expression was restricted to CD86^+^ B cells.

**Conclusions and clinical relevance:**

Thus, Tim1 and Tim3 are not essential for the induction of the type-2 response in lung allergy. This is contrary to what was proposed in a number of other studies using neutralizing and activating antibodies and questions the clinical relevance of Tim1 and Tim3 for novel allergy therapies.

*Cite this as*: J. L. Barlow, S. H. Wong, S. J. Ballantyne, H. E. Jolin and A. N. J. McKenzie, *Clinical & Experimental Allergy*, 2011 (41) 1012–1021.

## Introduction

Asthma is a complex chronic, inflammatory lung disease and is the most common chronic childhood disease affecting developed nations. The Th2 cytokine gene cluster, located on human chromosome 5q31-35, is clearly an important genetic determinant in asthma susceptibility. Interleukin 4 (IL-4), IL-5, IL-9, and IL-13 have all been shown to be essential components of the asthmatic response, including airway hyperreactivity (AHR), mucus hypersecretion, and B cell isotype-switching to IgE [1–6]. However, mapping studies have also identified the *Tapr* (T cell and airway phenotype regulator) locus on mouse chromosome 11, linked to the Th2 cytokine gene cluster, which conferred susceptibility to ovalbumin (OVA)-induced AHR in an experimental mouse model of asthma [7]. Polymorphisms in Tim (T cell immunoglobulin mucin) 1 (*Havcr1*) and Tim3 (*Havcr2*), two members of a new gene family discovered within the *Tapr* locus, were found to correlate with AHR and Th2 cytokine production, although the relative importance of Tim1 and Tim3 to this phenotype was unclear. McIntire et al. [8] have since reported that atopic resistance can be correlated with hepatitis A virus seropositivity and an insertion mutation in human TIM1, previously identified as the hepatitis A virus receptor (HAVCR1), suggesting that TIM1 may provide a biological basis for the link between decreasing exposure to HAV and the increasing incidence of atopy in westernized society. Associations between a number of mutations in both TIM1 and TIM3 and atopic disease have been found in other human asthma studies [9, 10].

Administration of a Tim1-human immunoglobulin (Tim1HuIg) fusion protein, or ligation of Tim1, either by a proposed counter-ligand in the form of a Tim4HuIg fusion protein [11], or an agonistic anti-Tim1 (clone 3B3) monoclonal antibody [12], resulted in the costimulation of T cell activation and the enhancement of IL-4 and IFN-γ production by CD3^+^ T cells. *In vivo* administration of the anti-Tim1 antibody (clone 3B3) prevented the development of respiratory tolerance and increased pulmonary inflammation [13], suggesting that blocking Tim1 function may suppress type-2 responses such as those observed in allergic inflammation. Indeed, Encinas and colleagues showed a down-regulation of OVA-induced allergic inflammation following anti-Tim1 antibody (clone 222414) treatment. However, recent reports have demonstrated that the interactions of antibodies recognizing different epitopes on Tim1 can mediate diametrically opposite effects. Anti-Tim1 antibodies, distinguishing specific epitopes in both the immunoglobulin (Ig) region and the mucin domain, were shown to reduce lung eosinophilia and lung inflammation, whereas an antibody directed at a different epitope in the mucin domain actually exacerbated the allergic response [14]. Furthermore, even anti-Tim1 antibodies recognizing the same epitope (clones 3B3 and RMT1-10), but with different avidities for Tim1, have been reported to elicit opposite functional effects [15]. Administration of a neutralizing anti-Tim3 antibody (clone 8H7), although ineffective when administered during an OVA sensitization and challenge model of asthma, was able to induce a partial decrease in AHR and Th2 cytokine production following the transfer of OVA-specific *in vitro* differentiated Th2 cells [16].

Thus, studies to date leave an unclear picture as to the importance of Tim1 and Tim3 in allergic asthma responses. To clarify the functional requirement for Tim1 and Tim3, we have analysed Tim1- and Tim3-deficient mouse lines and assessed the potential roles of these molecules in an *in vivo* model of antigen-driven acute lung allergy.

## Materials and methods

### Targeted disruption of the mouse Tim1 and Tim3 genes in embryonic stem cells

The *Havcr1* (*Tim1*^−/−^) mice were generated as described previously [17]. The *Havcr2* (*Tim3*^−/−^) replacement vector was constructed to insert the neomycin resistance gene into exon 2 of the *Havcr2* gene. This deleted the nucleotides encoding 71 amino acids (Phe57 – Lys127) of the 281 amino acid coding sequence of the *Tim3* gene. The 4.1 kb 5′ arm of homology was generated using E14.1 129J embryonic stem (ES) cell DNA as a template with PCR primers 5′-CAGCAATGCGGCCGCGTAAGCCAAATAAACCCTTTCCTACCTAGC-3′ and 5′-GGACAGGATCCCTTGCCCCAGCACATAGGCACAAGT G-3′. The 2.0 kb 3′ homology arm was generated using RPCI21 PAC488-E20 as a template with PCR primers 5′-ATTAGAACTAGTATT AGACATCAAAGCAGGTGAGTAG-3′ and 5′-AATCCTACTAGTGGCCTATATGGTAGATAACTATATG-3′. The *Havcr2* targeting vector was linearized and electroporated into E14.1 129J ES cells and used to generate mice homozygous for the gene by standard methods. *Tim3*^−/−^ mice, and corresponding wild-type animals, were derived from the original chimeras. Sequencing of Tim1 and Tim3 transcripts from BALB/c and 129 sources identified no polymorphisms between these strains; thus, mice were backcrossed for six generations to the BALB/c strain before intercrossing to generate homozygous mice. Genotypes were screened by southern analysis or PCR using primers, 5′-TGGTAAGAATGCCTATCTGCC-3′ and 5′-TCTGTAGACCATATCCTGGAGTTC-3′. The oligonucleotide specific to the inserted neomycin cassette was 5′-CTATCAGGACATAGCGTTGGCTACC-3′. All mice were maintained in a specific pathogen-free environment in SABU/CBS facilities according to UK Home Office regulations.

### RNA preparation

RNA was prepared from naïve tissues and from *in vitro* differentiated T helper cells [17] using RNAzolB, according to the manufacturer's instructions (AMS Biotechnology, Oxford, UK). Tim3 RT-PCR primers were as follows: 5′-AGGTCATTGGAAAATGCTTATGTGTTTGAG-3′ and 5′-CAGTAGGTCCCATGGTCATCC-3′, 5′-CTGCTGCAGGATACAGTTCCC-3′ and 5′-GAAATTAAGGACTCTGGAGAAACGATCAGAAC-3′, 5′-GAAATTAAGGACTCTGGAGAAACGATC-3′ and 5′-GCTACGTCAACAGAAAGCAGCCATCC-3′. HPRT primers and conditions were as described previously [18].

### Flow cytometry

Lung cell suspensions, treated with 720 μg/mL collagenase D (Amersham, Bucks, UK), were analysed for the expression of Tim1 and Tim3 by flow cytometry using the FACScalibur (BD, Biosciences, Oxford, UK) and FlowJo analysis software. Biotin-conjugated anti-Tim1 (clone RMT1-4, eBioscience SanDiego, CA, USA), biotin-conjugated anti-Tim3 (clone 8B.2C12, eBioscience), and PE-conjugated streptavidin were used.

### Sensitization and allergen exposure

BALB/c mice (6–12 weeks) were sensitized by an intraperitoneal administration of OVA (20 μg/injection, endotoxin low – removed using Affiniti Pak Endotoxin Removing Gel Columns (Pierce, Rockford, IL, USA) according to the manufacturer's instructions) in PBS (endotoxin free phosphate buffered saline, Sigma-Aldrich Dorset, UK), complexed in a 1 : 1 ratio with alum (Imject®, Thermo Scientific, Loughborough, UK), at days 0 and 12. Control mice received PBS only with alum. Aerosol administration of 1% OVA was undertaken on days 19, 20, and 21 for 20 min/day. Control animals received PBS.

### Measurement of airway resistance

On day 22, animals were analysed using invasive lung function measurements (EMMS, Bordon, UK) to assess AHR. Animals were anaesthetized, tracheostomized, and placed on a ventilator (MiniVent 845 ventilator, EMMS) at a rate of 150 breaths/min, with a tidal volume of 0.15 mL. After recording stable baseline pulmonary resistance for 3 min, increasing concentrations of acetyl-β-methylcholine chloride (methacholine) (Sigma-Aldrich) were administered by aerosol for 10 s at each concentration using an ultrasonic nebulizer, and pulmonary resistance was recorded for a 3-min period. eDaq software was used to analyse airway resistance.

### Tissue collection

Lungs were fixed in formalin (10% formaldehyde in a 0.9% saline solution) and stained with Giemsa, for inflammatory infiltrate, and periodic acid-Schiff (PAS) for mucus-producing goblet cells. Airway mucus production and numbers of infiltrating cells around blood vessels were measured blind using numerical scoring systems. Mediastinal lymph node cells were re-stimulated with 100 μg/mL OVA or 1 μg/mL purified anti-CD3 and cell supernatants were taken at a 72 h time-point. BAL fluid was collected, according to previously described protocols [19].

### Differential white blood cell counts and ^3^[H]-thymidine cell proliferation assay

Blood smears were stained with Giemsa (Sigma-Aldrich), following the manufacturer's instructions. Differential cell counts were made blind on 100 white blood cells per slide under oil and at a × 63 magnification. Blood was incubated at 4 °C overnight and serum was collected by centrifugation. Measurement of cell proliferation was undertaken as described previously [17] with or without 30 μg/mL IgM (AffiniPure F(ab′)_2_, μ chain specific, Jackson Laboratories, Bar Harbor, ME, USA).

### Cytokine and serum immunoglobulin enzyme linked immunosorbent assays

Cytokine ELISA utilized the sandwich format with capture and detection antibodies purchased from Becton Dickinson. ELISAs were performed according to the Becton Dickinson ELISA protocol. IL-13 was measured using the Quantikine ELISA kit (R&D Systems, Abingdon, UK). OVA-specific serum Ig ELISAs were undertaken using immunosorbent plates coated with 5 μg/mL OVA, overnight, and then as described previously [17].

### Statistical analysis

The significance of the differences between experimental groups was analysed using a two-way anova with Bonferroni post-test (for dose response to methacholine) or the Student unpaired *t*-test.

## Results

### Allergic lung inflammation can be induced in the absence of Tim1

**Fig. 1:**
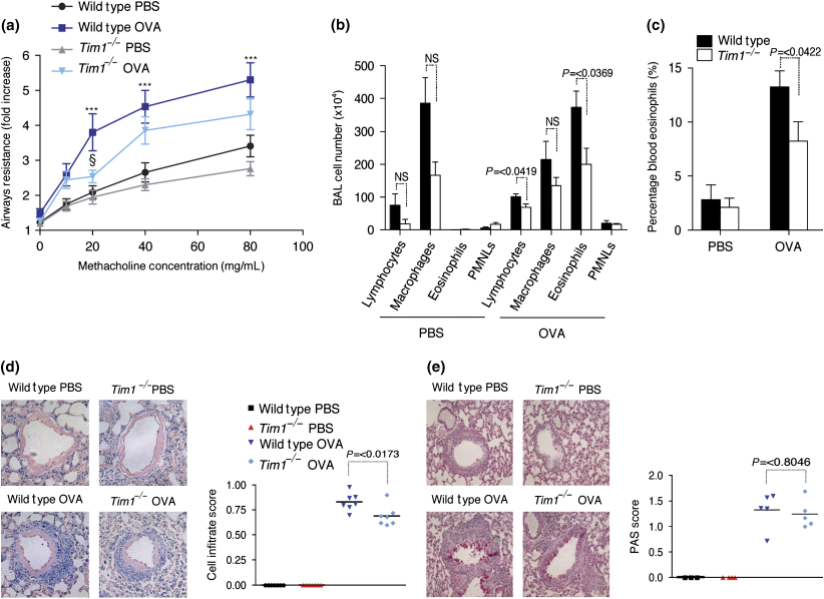
(a) Airway resistance in wild type and *Tim1*^−/−^ mice measured using invasive plethysmography. Results are shown for lung resistance above the baseline reading. ^***^P=<0.001 between wild-type PBS and ovalbumin (OVA)-treated mice. §*P*=<0.05 between wild-type and *Tim1*^−/−^ OVA-treated mice. (b) Differential cell counts were performed on the BAL fluid in 250 μL from wild-type and *Tim1*^−/−^ mice. (c) The percentages of eosinophils in peripheral blood were assessed by differential cell counts. (d) Cellular infiltration and (e) mucus production determined by analysis of lung sections stained with Giemsa or periodic acid-Schiff, respectively. × 20 magnification. Data are representative of two to three repeat experiments (*n*=8–10).

Airway obstruction is the most debilitating symptom of asthma and is a result of excessive contraction of smooth muscle. Bronchoconstriction can be determined during allergic lung inflammation using invasive plethysmography to directly measure airway resistance in response to lung agonists, e.g. methacholine. To investigate a role for Tim1 in allergic disease, wild type and *Tim1*^−/−^ mice were first sensitized intraperitoneally and then challenged in the lung with aerosolized OVA to induce a type-2 mediated inflammatory response. As expected, the airway resistance in OVA-treated wild-type mice increased compared with PBS-treated control mice (Fig. 1a). *Tim1*^−/−^ mice displayed a minor, although statistically significant, reduction in airway resistance at the 20 mg/mL methacholine dose, but overcame this deficit at higher doses (40–80 mg/mL).

We also investigated the requirement for Tim1 during the allergic lung inflammatory response. The total cell counts from the bronchoalveolar lavage (BAL) showed that the numbers of lymphocytes, macrophages, eosinophils, and polymorphonuclear leucocytes (PMNLs) were equivalent in the PBS-treated *Tim1*^−/−^ and wild-type mice (Fig. 1b). OVA treatment led to a minor, but statistically significant, decline in lymphocyte and eosinophil cell numbers in the BAL of *Tim1*^−/−^ mice, as compared with wild-type mice (Fig. 1b). However, eosinophils, the main infiltrating cell, still increased significantly in OVA-treated *Tim1*^−/−^ mice compared with PBS-treated control mice (Fig. 1b).

To explore whether the reduction in infiltrating cells resulted from an inability to migrate or home efficiently, or from a deficit in circulating eosinophils, we examined blood cell composition. Differential blood cell counts of lymphocytes, monocytes, eosinophils, and PMNLs were performed on samples from the PBS- and OVA-challenged mice. Blood eosinophils constituted ∼2% of leucocytes in wild-type and *Tim1*^−/−^ PBS control mice. In wild-type OVA-treated mice, this was increased to around 12%. However, OVA-treated *Tim1*^−/−^ mice failed to develop equivalently severe eosinophilia (Fig. 1c). No other blood cell types were altered by the Tim1 deficiency (data not shown). The coincident decrease in the levels of circulating eosinophils and lung infiltrating eosinophils implies that the deficit in cellular infiltrate arises from the reduction in circulating cells rather than an inability to migrate to the lungs.

The deficit in cellular infiltrate observed in the BAL of *Tim1*^−/−^ mice was also mirrored in the lung tissue. Here, the perivascular cell infiltrate score was again significantly lower, but not completely abrogated, in the *Tim1*^−/−^ lungs (Fig. 1d). It is not clear whether this relates to a decrease specifically in lung eosinophils. Mucus production, identified by PAS staining of airway epithelium, was similar in the OVA-treated wild type and *Tim1*^−/−^ groups (Fig. 1e).

**Fig. 2:**
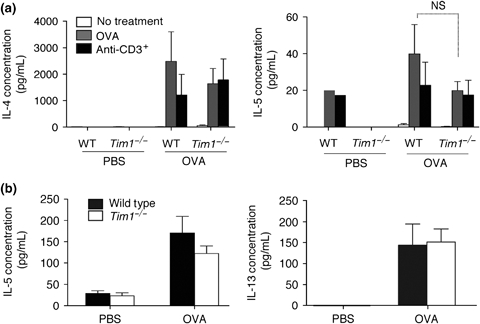
Ovalbumin (OVA)-specific and polyclonal cytokine production was determined from (a) restimulated mesenteric lymph node cell cultures and (b) BAL fluid samples by sandwich ELISA. Data are representative of two repeat experiments (*n*=8–10).

As Tim1 is reportedly expressed on CD4^+^ Th2 cells, we investigated the proliferation and cytokine responses in OVA-restimulated cells cultured from the draining mediastinal lymph nodes. Cells from OVA-challenged mice produced very little antigen-specific IL-2 and no IFN-γ (data not shown), but elevated levels of the Th2-cytokines IL-4 and IL-5 (Fig. 2a). The levels of IL-5 were slightly reduced in the *Tim1*^−/−^ samples, but this did not attain statistical significance. Although IL-4 could not be detected in the BAL fluid, OVA-induced IL-5 and IL-13 levels were found to be normal in *Tim1*^−/−^ mice (Fig. 2b). The proliferation of mediastinal lymph node cells from wild-type and *Tim1*^−/−^ mice, in response to OVA, also demonstrated no appreciable differences between the OVA-challenged groups (data not shown). Collectively, these data show that OVA-induced T cell proliferation and cytokine responses do not appear to be significantly affected by Tim1 deficiency during the type-2-driven allergic lung response.

### Tim1 is predominantly expressed on activated B cells, not T cells during allergic lung inflammation

**Fig. 3:**
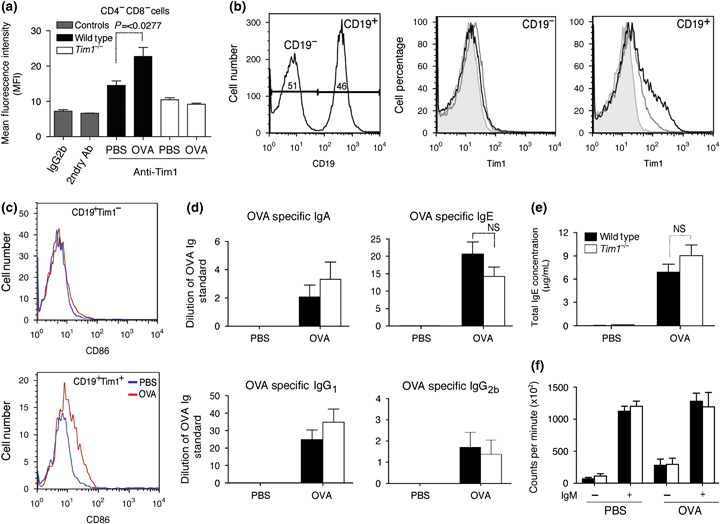
(a) Mean fluorescence intensity of Tim1 expression on CD4^−^CD8^−^ in the lung as assessed by flow cytometry. (b) Left panel, histogram of CD19^−^ and CD19^+^ gated populations in the lung. Middle and right panel, Tim1 expression within the CD19^−^ gate and CD19^+^ gate, respectively, in the lung. Grey line, PBS -treated mice; black line, ovalbumin (OVA) mice; filled grey, isotype control. (c) OVA-specific IgA, IgE, IgG_1_, and IgG_2b_, and (d) total IgE production in serum. (e) Mediastinal lymph node cell proliferation in response to media only or IgM, measured by the incorporation of ^3^[H]-thymidine. Data are representative of two repeat experiments (*n*=8–10).

The lack of a demonstrable effect on type-2 cytokine production is at odds with the existing dogma regarding Tim1 bioactivity and its reported expression on Th2 cells. Thus, we undertook extensive studies to demonstrate convincing staining for Tim1 on the surface of naïve T cells or differentiated Th1 or Th2 cells. During our analysis of the cell surface expression of Tim1, we used the anti-Tim1 antibody RMT1-4 (16) due to it consistently recognizing Tim1 as assessed by its binding to native Tim1, transgenically expressed Tim1 of either the BALB/c or the C57BL/6 allotype (data not shown), and its failure to stain cellular products in the Tim1-deficient mice (Fig. 3a).

Significantly, we were unable to find any detectable Tim1 expression by flow cytometric analysis on CD3^+^CD4^+^ or CD3^+^CD8^+^ T cell populations in either naïve or allergic lung tissue (data not shown). However, further investigation of the expression pattern of Tim1 showed that it was detectable on CD4^−^CD8^−^ cells, and restricted within that population to CD19^+^ B cells from naïve lung tissue (Fig. 3a and b). We found no evidence for Tim1 expression on any other cell population at this time-point. Furthermore, Tim1 was up-regulated on B cells after induction of allergic lung inflammation (Fig. 3b). Additionally, further phenotypic analysis indicated that the Tim1 expression during lung allergy coincides with CD86 expression on the B cells, suggesting that it is expressed on activated B cells capable of co-stimulation of T cell activation (Fig. 3c). Although B cells have not been shown to be essential for allergic lung inflammation, the expression of Tim1 on CD19^+^CD86^+^ cells led us to investigate whether *Tim1*^−/−^ mice had defects in B cell function following the induction of lung allergy. To test this, the production of OVA-specific serum Igs was analysed. In wild-type mice, OVA treatment induced a large increase in OVA-specific IgG_1_ and IgE antibodies, and some IgA and IgG_2b_ antibodies, as well as an increase in total IgE production (Fig. 3d and e). No defects were found in serum antibody production during allergic lung inflammation in *Tim1*^−/−^ mice. Similarly, the proliferative response of B cells from *Tim1*^−/−^ mice following IgM stimulation *in vitro* was also found to be normal (Fig. 3f). Therefore, B cell function was not found to be affected by deficiency in Tim1.

### Tim3 is expressed on CD11c^+^ myeloid cells and CD4^+^CD25^+^ T cells during allergic lung inflammation

Tim3, like Tim1, has also been associated with a number of human diseases including multiple sclerosis and rheumatoid arthritis, and has been shown to be expressed on CD4^+^ Th1 cells in mice. Components of the type-1 immune response have previously been shown to affect the allergic lung response. To address the potential role of Tim3 in experimental asthma, we developed a mouse line deficient for *Tim3* (*Havcr2*) by gene targeting in ES cells (supporting information Fig. S1). *Tim3*^−/−^ mice were healthy and displayed no overt phenotypic abnormalities.

**Fig. 4:**
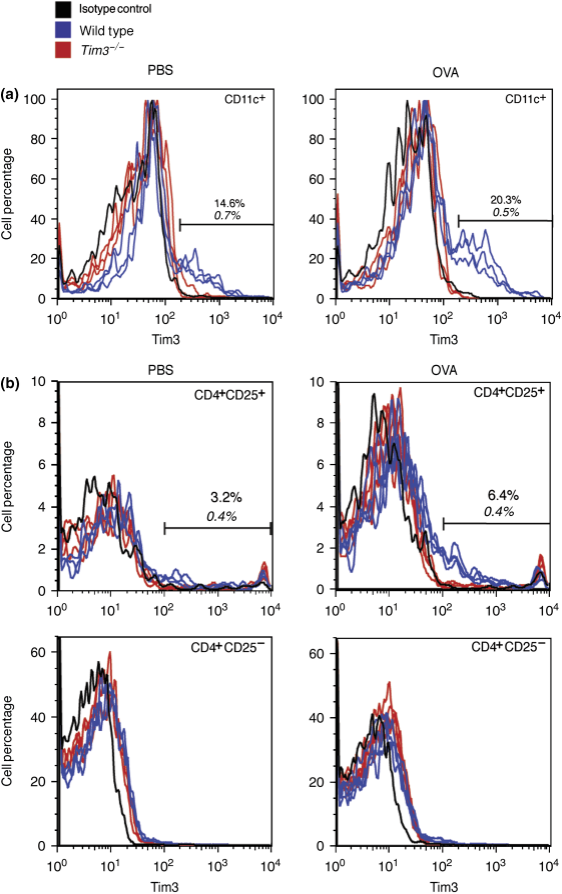
(a) Histograms showing Tim3 expression on CD11c^+^ gated cells and (b) T cells in the lungs of mice treated with PBS or ovalbumin (OVA). T cells were gated as being CD4^+^CD25^+/−^ by flow cytometry. Average percentages for three mice per group are shown. Regular text, wild type; italic text, *Tim3*^−/−^. Data are representative of two repeat experiments.

We first wanted to ascertain Tim3 expression during the allergic response. Lung tissue was taken from wild-type and *Tim3*^−/−^ mice, in which lung allergy had been induced. Tim3 expression in these mice was compared with that in PBS control mice. No differences in the percentage of CD4^+^ or CD8^+^ T cells, CD19^+^ B cells, or CD11c^+^ alveolar macrophages were observed between wild-type and *Tim3*^−/−^ mice (data not shown). Tim3 was expressed in naïve wild-type mice only on CD11c^+^ lung cells (Fig. 4a). The expression of Tim3 was increased on CD11c^+^ cells in OVA-treated mice. Although CD11c is normally considered to be a surface marker for dendritic cells, in the lung, it is expressed mostly on alveolar macrophages. Tim3 expression was also found on CD4^+^CD25^+^ cells. Notably, these cells were only observed in OVA-treated mice (Fig. 4b). Thus, although Tim3 has been associated in previous reports with type-1 immunity, our data show that Tim3 is up-regulated during the type-2 response in allergic lung inflammation.

### Tim3 is not required for the induction of allergic lung inflammation

**Fig. 5:**
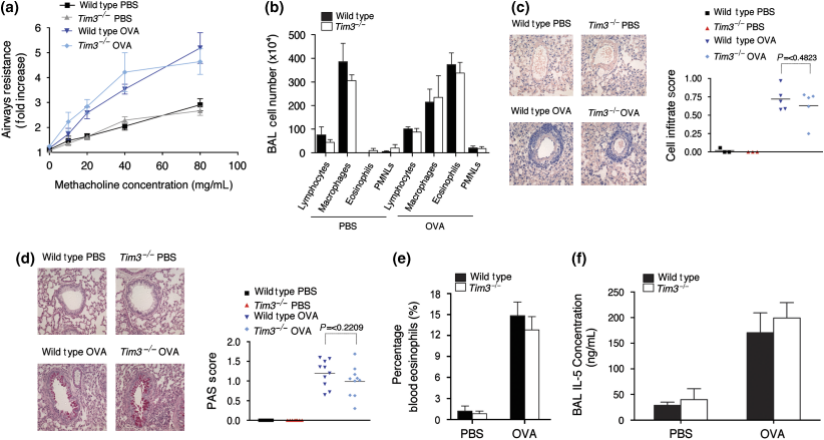
(a) Airway resistance in wild-type and *Tim3*^−/−^ measured using invasive plethysmography. Results are shown for lung resistance above the baseline reading. (b) Differential cell counts were performed on the BAL fluid in 250 μL from wild-type and *Tim3*^−/−^ mice. (c) Cellular infiltration and (d) mucus production determined by analysis of lung sections stained with Giemsa or PAS, respectively. × 20 magnification. (e) The percentages of eosinophils in peripheral blood were assessed by differential cell counts. (f) IL-5 production in the BAL fluid as measured by ELISA. Data are representative of two to three repeat experiments (*n*=8–10).

As Tim3 was expressed in the lung during allergic responses, we next wanted to test its functional significance in those responses. We were unable to measure any detectable differences in airway resistance, compared with the OVA-treated wild-type mice, caused by the absence of Tim3 (Fig. 5a).

In agreement with the airway resistance data, the absence of Tim3 also exerted no detectable effect on the lung inflammatory response. Cellular infiltration into the lung, as assessed by BAL cell counts and lung histology, was comparable to wild-type controls (Fig. 5b and c). Furthermore, blood eosinophilia and airway mucus production were also found to be normal in the Tim3-deficient mice, as were type-2 cytokine responses in the BAL fluid, as measured by IL-5 production (Fig. 5d–f). Cytokine production from draining lymph node cells, and serum Ig production, including OVA-specific IgE production, were also unchanged by the absence of Tim3 (data not shown).

## Discussion

Using *Tim1*^−/−^ and *Tim3*^−/−^ mice, we have investigated the role of these molecules in the regulation of type-2 responses during lung allergy. Although previous reports have implicated both Tim1 and Tim3 as being important components of the T cell cytokine response, we now demonstrate that Tim1 and Tim3 are dispensable in regulating AHR in a mouse model of allergic asthma.

Th2 cell responses seem to remain largely intact in the absence of Tim1 and Tim3, although in *Tim1*^−/−^ mice, there are minor, but statistically significant, reductions in lung function measurements at low methacholine concentrations, blood eosinophilia, and lung cell infiltration. It is unclear what underlies these small defects, but does suggest that Tim1 is largely not required for the response to proceed. We were unable to detect a statistically significant deficit in OVA-specific IL-5 production from the draining lymph node cells, or BAL fluid, from *Tim1*^−/−^ mice. However, it remains possible that levels of IL-5 and eotaxin, or other granulocyte growth factors such as GM-CSF, may be suppressed at other time-points in the response, thereby underlying this deficit in circulating eosinophils.

Encinas et al. [13] previously demonstrated, using an anti-Tim1 mAb (clone 222414), that pulmonary inflammation, and IL-10 and IL-13 levels were reduced following anti-Tim1 administration, suggesting that Tim1 was required to induce normal allergic lung inflammation. Although we observed minor reductions in eosinophil infiltration, we did not detect a significant reduction in type-2 cytokine production from cells isolated from the draining lymph nodes and Tim1 was not essential for AHR or substantial lung inflammation.

One striking observation made from our analysis of naïve mice, using Tim1-deficient mice as negative controls for the cell surface staining of Tim1, was the predominant expression of Tim1 on the surface of a B cell population, and not T cells during lung allergy. Although the Tim1 transcript has been reported previously in B cell cDNA from both the spleen and the lymph nodes in BALB/c and C57BL/6 mice, and Tim1 expression has been noted on activated B cells, further characterization *in vivo* was not undertaken in the lung [17]. We found that Tim1^+^ B cells were characterized as CD19^+^CD86^+^ B cells, probably representing a more activated B cell population. Moreover, following *in vitro* B cell activation with the polyclonal stimulator anti-IgM, Tim1 was found to be expressed on almost all proliferating B cells, highlighting the regulation of Tim1 expression on activated B cells [17]. By contrast, we failed to observe the regulation of Tim1 on T cells, although it is possible that Tim1 is expressed on T cells during other types of immune response.

The role of up-regulated Tim1 in B cells remains uncertain, but the fact that it is induced by activation through the B cell receptor, but not via LPS stimulation [17], indicates that Tim1 is differentially regulated depending on the antigen encountered and that it may play a role in forming these divergent responses. Following lung challenge, while flow cytometric analysis failed to identify Tim1^+^ T cells in the lungs, CD19^+^Tim1^+^ B cells were identified and these were also CD86^+^. Thus, Tim1 appears to be a marker of a subset of activated B cells during *in vivo* immune responses to antigen. Despite the expression of Tim1 on B cells, we found no alteration in Ig expression or B cell proliferation. Wong and colleagues also failed to find a role for Tim1 on B cells in antigen presentation in the spleen.

We also found no measurable role for Tim3 in this OVA-induced model of allergic asthma, although unlike Tim1, it was expressed on a population of CD4^+^CD25^+^ cells, which would classically be considered to be either T regulatory cells or activated T cells. Our results contrast with the report by Kearley et al. [16], in which OVA-induced airway inflammation was reduced following treatment with an anti-Tim3 antibody and correlated with a decrease in IL-5 expression. However, these differences were only observed in a model involving the transfer of *in vitro* generated Th2 cells into naïve recipients, and not in a more conventional OVA-sensitization model of airway inflammation [16]. Thus, although the modulation of Tim3 can have an impact on a type-2 response, it is likely that the effects of inhibiting Tim3 vary considerably depending on the model used.

Our results were somewhat unexpected, given previous reports regarding Tim1, suggesting not only that the genes encoding Tim1 and Tim3 within the *Tapr* locus, along with their associated polymorphisms, may play a role in regulating pulmonary function [7] but that anti-Tim1 and anti-Tim3 antibodies administered *in vivo* exerted wide-ranging effects on T cell function during diabetes [20], transplant rejection [20], pulmonary inflammation [12], and EAE [21]. These differences may reflect the variation in the biological effects that have been reported for the interaction of anti-Tim1 antibodies with agonistic and antagonistic epitopes on Tim1 [14].

Furthermore, it is possible that there is a functional redundancy between the Tim family members that also include Tim2, Tim4, Tim5, and Tim 6. Certainly, Tim2-deficiency has been shown to exacerbate the allergic inflammatory response due to the up-regulation of type-2 cytokine release by T cells [22]. However, the roles of the other family members in such responses are unknown.

Genetic association studies in humans have indicated that polymorphisms in HAVCR1 correlate with the development of atopic disease [8, 23]. Similar studies have also failed to find any such association in a Japanese population [24]. We now demonstrate that although *Tim1*^−/−^ mice show a slight decrease in AHR, which probably arises as a result of a broad-spectrum reduction in lung cell infiltrates, notably eosinophils, the absence of Tim1, or indeed Tim3, does not prevent the allergic lung response, suggesting that they may not be clinically relevant targets for new therapies.

## Supporting Information

**Fig. S1.** Inactivation of *Havcr2* (*Tim3*) by homologous recombination (A) The structure of the *Tim3* locus, the targeting vector, and predicted homologous recombination event. (B) Genotyping of genomic DNA from *Tim3* genetargeted mice by PCR analysis. (C) RT-PCR analysis of Tim3 expression, assessed using exon-specific primer sets in wild type and *Tim3*^−/−^ Th1 and Th2 cells. Con, vector containing full length Tim3 transcript; Neg, negative control. (D) Flow cytometric analysis of Tim3 expression on lung cell suspensions from OVA sensitised wild type and *Tim3*^−/−^ mice.
