# Supplementary figures and images for: Tim1 and Tim3 are not essential for experimental allergic asthma
Source: Clin Exp Allergy. 2011 Jul;41(7):1012–21. doi: 10.1111/j.1365-2222.2011.03728.x (PMC3132443; doi:10.1111/j.1365-2222.2011.03728.x)

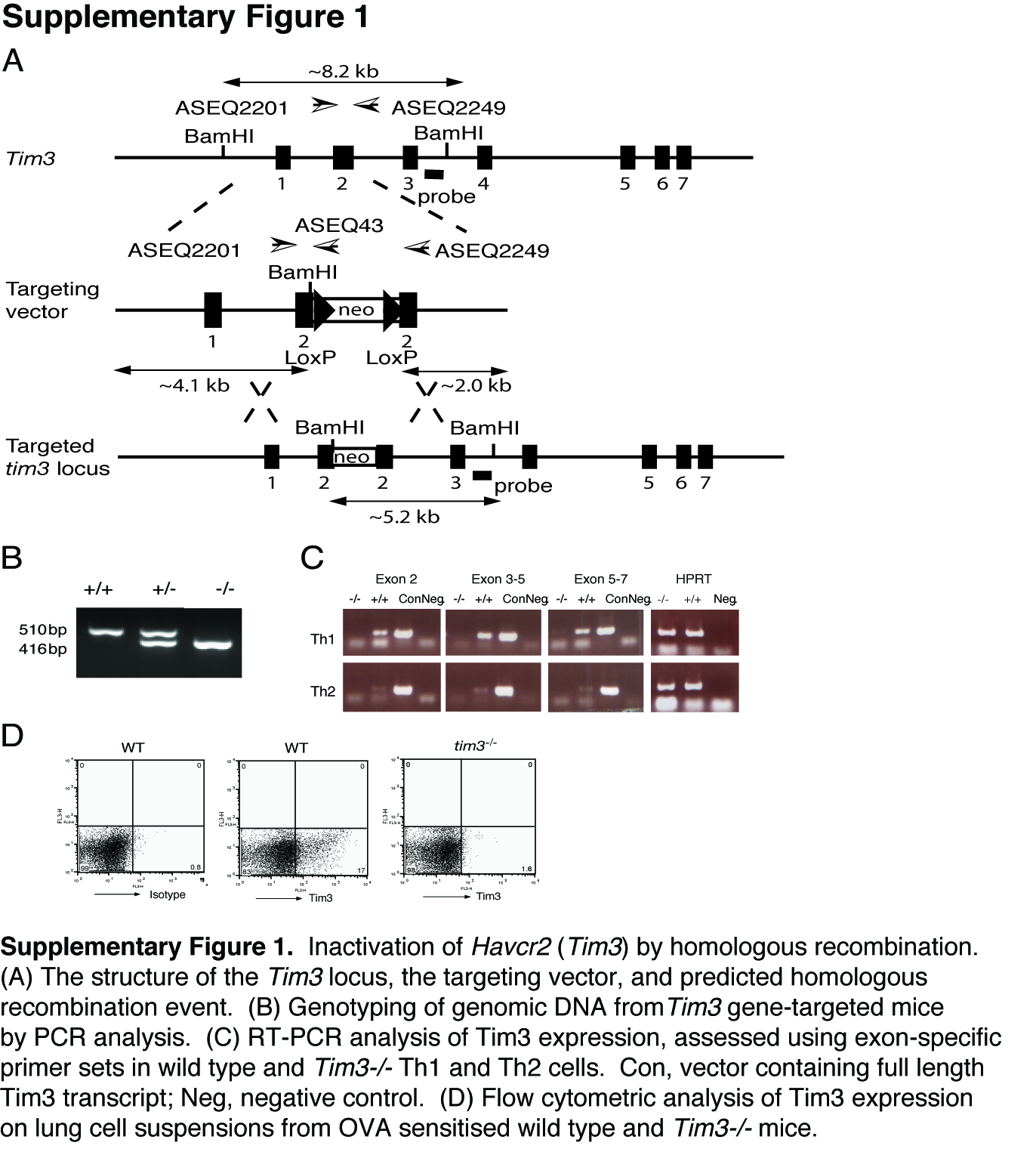

Supplement: Supplementary file 1 [file cea0041-1012-SD1.tif]
